# Prioritization of nasal polyp-associated genes by integrating GWAS and eQTL summary data

**DOI:** 10.3389/fgene.2023.1195213

**Published:** 2023-06-23

**Authors:** Masahiro Yoshikawa, Kensuke Asaba, Tomohiro Nakayama

**Affiliations:** ^1^Division of Laboratory Medicine, Department of Pathology and Microbiology, Nihon University School of Medicine, Tokyo, Japan; ^2^Technology Development of Disease Proteomics Division, Department of Pathology and Microbiology, Nihon University School of Medicine, Tokyo, Japan; ^3^Department of Computational Diagnostic Radiology and Preventive Medicine, The University of Tokyo Hospital, Tokyo, Japan

**Keywords:** nasal polyps, genome-wide association studies, expression quantitative trait locus, gene prioritization, summary data-based Mendelian randomization, Bayesian colocalization

## Abstract

**Background:** Nasal polyps (NP) are benign inflammatory growths of nasal and paranasal sinus mucosa that can substantially impair patients’ quality of life by various symptoms such as nasal obstruction, insomnia, and anosmia. NP often relapse even after surgical treatment, and the curative therapy would be challenging without understanding the underlying mechanisms. Genome wide association studies (GWASs) on NP have been conducted; however, few genes that are causally associated with NP have been identified.

**Methods:** We aimed to prioritize NP associated genes for functional follow-up studies using the summary data-based Mendelian Randomization (SMR) and Bayesian colocalization (COLOC) methods to integrate the summary-level data of the GWAS on NP and the expression quantitative trait locus (eQTL) study in blood. We utilized the GWAS data including 5,554 NP cases and 258,553 controls with 34 genome-wide significant loci from the FinnGen consortium (data freeze 8) and the eQTL data from 31,684 participants of predominantly European ancestry from the eQTLGen consortium.

**Results:** The SMR analysis identified several genes including TNFRSF18, CTSK, and IRF1 that were associated with NP due to not linkage but pleiotropy or causality. The COLOC analysis strongly suggested that these genes and the trait of NP were affected by shared causal variants, and thus were colocalized. An enrichment analysis by Metascape suggested that these genes might be involved in the biological process of cellular response to cytokine stimulus.

**Conclusion:** We could prioritize several NP associated genes including TNFRSF18, CTSK, and IRF1 for follow-up functional studies in future to elucidate the underlying disease mechanisms.

## 1 Introduction

Nasal polyps (NP) are typically bilateral inflammatory growths of nasal and paranasal sinus mucosa that occur in up to 4% of the adult population (Stevens et al., 2014). Although NP are non-malignant and may be asymptomatic, they are mostly complicated with chronic rhinosinusitis (CRS) and can impair the quality of life by causing nasal obstruction and congestion, nasal discharge, facial pain, insomnia, and anosmia (Hopkins, 2019; Ta, 2019). Surgical intervention must be considered in patients whose symptoms are not ameliorated or controlled with the short-term use of systemic glucocorticoids and long-term use of inhaled glucocorticoids; however, rates of polyp recurrence are high (Hopkins, 2019). Genetic factors may play a role in the pathogenesis (Hopkins, 2019), but the curative therapy would be challenging without elucidating the underlying disease mechanisms (Ta, 2019).

Genome-wide association studies (GWASs) can identify genome-wide significant loci with genetic variants associated with a trait of interest; however, elucidating the molecular mechanisms underlying this association is difficult. Some variants do not change the amino acid sequences but the expression of genes, and other variants are in linkage disequilibrium (LD) with truly causative variants (Zhu et al., 2016). Kristjansson et al. (2019) conducted a GWAS on NP with 4,366 cases by meta-analyzing two datasets from Iceland and the United Kingdom and found 10 genome-wide significant loci associated with NP. Of these 10 loci, rs34210653 and rs1050152 are missense variants in ALOX15 and SLC22A4 genes, respectively. However, rs174535 is synonymous, and the other seven loci are located in intronic or intergenic regions. The non-coding regions can regulate transcriptional activities of genes, and there are examples of causative genes that are distant from genome-wide significant loci (Zhu et al., 2016). Therefore, it is a non-trivial task to connect these non-coding variants to affected genes for the functional interpretation of GWAS results in “the post-GWAS era,” and thus, many bioinformatical methods for gene prioritization have been developed to fill the gap (Li and Ritchie, 2021). In this study, we used two gene prioritization methods, namely, summary data-based Mendelian randomization (SMR) (Zhu et al., 2016) and Bayesian colocalization (COLOC) analyses (Giambartolomei et al., 2014), to integrate the summary-level data of the GWAS on NP and the expression quantitative trait locus (eQTL) data in blood. As a result, we prioritized several NP-associated genes for follow-up functional studies in future to elucidate the underlying disease mechanisms.

## 2 Methods

### 2.1 Datasets

For the summary-level data of the GWAS on NP, we used summary statistics of 5,554 cases with NP and 258,553 controls from the FinnGen consortium data freeze 8 (released on 1 December 2022). To the best of our knowledge, these are the latest GWAS data with the largest number of NP cases to date. FinnGen aims to collect and analyze the genome and national health register data of 500,000 Finnish individuals (Kurki et al., 2023). NP were defined as International Classification of Diseases (ICD)-10 code J33, ICD-9 code 471, and ICD-8 code 505. FinnGen identified a total of 34 loci that were associated with NP at *p* < 5.0 × 10^−8^ within a ± 500 kb window, none of which was located in an exon (Supplementary Table S1). For the eQTL data, we used summary-level data for blood-derived gene expression from 31,684 participants (25,482 samples were from whole blood and 6,202 were from peripheral blood mononuclear cells) of predominantly European ancestry identified by the eQTLGen consortium (Võsa et al., 2021). The GWAS summary statistics for the prioritized gene expression from the eQTLGen consortium and for eosinophil cell count in the European population from the Blood Cell Consortium were available from the MRC IEU OpenGWAS database (Hemani et al., 2023). For the splicing quantitative trait locus (sQTL) data, we used summary-level data from 670 whole-blood samples of mostly European ancestry identified by the GTEx project (GTEx Consortium, 2020).

### 2.2 SMR analysis

We conducted SMR and heterogeneity in dependent instruments (HEIDI) tests in cis regions using the SMR software tool version 1.03. Detailed methods for SMR analysis were described in the original SMR paper (Zhu et al., 2016). In brief, SMR analysis utilizes a well-established MR method (Hemani et al., 2023) using a single-nucleotide variant (SNV, also known as a single-nucleotide polymorphism [SNP]) at a top cis-eQTL as an instrumental variable (IV), an effect from summary-level eQTL data as exposure, and an effect from summary-level GWAS data for a trait of interest as an outcome, to investigate a causal or pleiotropic association (where the same causal variant is shared) between the gene expression and the trait. The SMR method cannot distinguish a causal association (where the gene expression causally mediates the trait) from a pleiotropic association (where the same SNV affects both the gene expression and trait) because the MR method with a single IV cannot distinguish causality from pleiotropy. However, the HEIDI test can distinguish causality and pleiotropy from linkage (where two different SNPs in LD separately impact the gene expression and the trait), which is of less biological interest than causality and pleiotropy. We also conducted SMR and HEIDI tests using summary-level sQTL data as exposure. To conduct the SMR and HEIDI tests, we used the default settings in the SMR software tool. In particular, the *p*-value threshold to select the top associated eQTL or sQTL for the SMR test was 5.0 × 10^−8^, and a window around the center of the probe to select cis-eQTLs or cis-sQTLs was 2 Mb. By default, we only conducted SMR analysis in cis regions. For the SMR tests, a *p*-value below 2.59 × 10^−6^ (0.05 divided by 19,250 probes in the eQTL data by Bonferroni correction) or 7.67 × 10^−7^ (0.05 divided by 65,127 probes in the sQTL data) was considered statistically significant. For the HEIDI test, a *p*-value below 0.05 was considered significant, indicating that the observed association was due to linkage (Zhu et al., 2016).

### 2.3 COLOC analysis

We conducted COLOC analysis using the coloc package in R software (version 4.0.3) (Giambartolomei et al., 2014). COLOC analysis assesses whether SNVs associated with gene expression and phenotype at the same locus are shared causal variants, and thus, gene expression and phenotype are “colocalized.” COLOC analysis calculates posterior probabilities (PPs) of the five hypotheses: 1) H0; no association with either gene expression or phenotype; 2) H1; association with gene expression, not with the phenotype; 3) H2; association with the phenotype, not with gene expression; 4) H3; association with gene expression and phenotype by independent SNVs; and 5) H4; association with gene expression and phenotype by shared causal SNVs. A large PP for H4 (PP.H4 above 0.75) strongly supports shared causal variants affecting both gene expression and phenotype (Giambartolomei et al., 2014). We assigned a prior probability of 1 × 10^−4^ for H1 and H2 and a prior probability of 1 × 10^−5^ for H4 as the default settings of the coloc.abf function. We tested the region within 1 Mb on either side of the lead variant with the smallest *p*-value at the region in the GWAS data.

### 2.4 Enrichment analysis

We submitted the gene symbols to the Metascape web portal (Zhou et al., 2019) (https://metascape.org/gp/index.html#/main/step1) using “express analysis” with default settings.

### 2.5 Mediation analysis by Mendelian randomization

Using two-sample Mendelian randomization (MR) and inverse variance weighted (IVW) multivariable MR analyses, we conducted mediation analysis (Moncla et al., 2022) to investigate whether the effect of the prioritized gene expression on NP risk was mediated by another disease or trait. Two-sample MR and IVW multivariable MR analyses were conducted using the TwoSampleMR package (version 0.5.6) in R software (version 4.0.3), as described previously (Yoshikawa et al., 2022). First, we conducted two analyses of univariable two-sample MR that estimated the causal effects of the prioritized gene expression on NP risk and eosinophil cell count. The SNVs were selected from the exposure dataset as instrumental variables (IVs) that were associated with the significant exposure (*p* < 5.0 × 10^−8^) and were not in LD (r^2^ < 0.001 and distance >10,000 kb) with the other SNPs. We excluded IV SNVs from the analysis, if any, that were associated with the outcome at *p* < 5.0 × 10^−8^. The F-statistic for each IV SNV was calculated (Shim et al., 2015). IVs with an F-statistic below 10 are considered weak instruments (Burgess et al., 2017). The IVW method was used as the main analysis of the two-sample MR study, followed by a series of sensitivity analyses including the weighted median method, weighted mode method, MR-Egger intercept, Cochran’s Q statistic calculation for the IVW method, and MR-PRESSO global test (using the run_mr_presso function). When Cochran’s Q statistic indicated the presence of heterogeneity among IV SNVs (*p* < 0.05), we used a multiplicative random-effects model for the IVW method. Otherwise, we used a fixed-effects model. Next, we conducted IVW multivariable MR analysis using the prioritized gene expression and eosinophil cell count as exposures and NP risk as an outcome using the mv_ivw function.

## 3 Results

### 3.1 SMR and COLOC analyses using blood eQTL data

The overall results are presented in Table 1.

**TABLE 1:** SMR/HEIDI results using the GWAS data on NP and the blood eQTL data, and the COLOC results between the GWAS data and the blood eQTL data of the genes that passed the SMR test. Probe and SNV positions are written in GRCh37. Bold numbers mean significant P_SMR_ (<2.59 × 10^−6^), non-significant P_HEIDI_ (>0.05), and large PP.H4 (>0.75). Ch, chromosome; P_GWAS_, *p*-value of the top SNV from the GWAS data; P_eQTL_, *p*-value of the top SNP from the eQTL data; P_SMR_, *p*-value for the SMR test; B_SMR_, effect size from the SMR test; SE_SMR_, standard error of the B_SMR_; P_HEIDI_, *p*-value for the HEIDI test; N_HEIDI_, the number of SNVs used in the HEIDI test; GWAS SNV, lead variant with the smallest *p*-value from the GWAS data in the region analyzed by the colocalization test (±1 Mb from the GWAS SNV position); N_SNV_, the number of SNVs used in the colocalization test.

			SMR and HEIDI tests					COLOC test		
Ch	**Gene**	Gene probe	Top SNV	P_GWAS_	P_SMR_	B_SMR_	P_HEIDI_	GWAS SNV	PP.H4	N_SNV_
		Probe position	SNV position	P_eQTL_		SE_SMR_	N_HEIDI_	SNV position	PP.H3	
1	**TNFRSF18**	ENSG00000186891	rs3813201	1.7e-06	**2.4 × 10** ^**−6**^	−0.33	**0.34**	rs3753347	**0.968**	920
		1140479	1151232	6.8e-172		0.070	20	1143451	0.031	
1	**CTSK**	ENSG00000143387	rs2089081	1.6e-11	**1.9 × 10** ^**−11**^	0.21	**0.65**	rs2089081	**0.759**	2612
		150774741	150800117	3.3e-310		0.031	20	150800117	0.241	
2	**MIR4772**	ENSG00000264764	rs6543133	1.5e-11	**5.9 × 10** ^**−10**^	−0.38	4 × 10^−9^	rs4851011	0.005	800
		103048787	103040177	1.2e-54		0.062	20	103089678	0.995	
2	**AC007278.2**	ENSG00000236525	rs10206291	1.4e-11	**3.7 × 10** ^**−11**^	−0.23	3 × 10^−12^	rs4851011	9e-05	1663
		103051108	103038863	1.2e-226		0.035	20	103089678	1.00	
2	**IL18RAP**	ENSG00000115607	rs6734762	1.4e-12	**1.5 × 10** ^**−12**^	−0.17	6 × 10^−12^	rs4851011	0.018	3071
		103052087	103062926	3.3e-310		0.024	20	103089678	0.982	
2	**AC007278.3**	ENSG00000234389	rs13021177	1.5e-12	**1.9 × 10** ^**−12**^	−0.13	5 × 10^−7^	rs4851011	0.396	2347
		103056054	103056493	3.3e-310		0.019	20	103089678	0.604	
5	**RAPGEF6**	ENSG00000158987	rs7731071	4.8e-09	**2.2 × 10** ^**−8**^	0.58	0.029	rs35260072	7e-06	1023
		130865271	130973483	1.5e-79		0.103	20	131630852	1.00	
5	**P4HA2**	ENSG00000072682	rs11955347	6.8e-14	**2.8 × 10** ^**−10**^	−1.55	3 × 10^−7^	rs35260072	0.178	1056
		131579269	131567924	1.1e-31		0.245	20	131630852	0.822	
5	**SLC22A5**	ENSG00000197375	rs11242109	7.4e-16	**9.3 × 10** ^**−16**^	0.24	0.014	rs35260072	0.691	3060
		131718375	131677047	3.3e-310		0.030	20	131630852	0.309	
5	**IRF1-AS1**	ENSG00000197536	rs7713065	1.2e-09	**1.9 × 10** ^**−7**^	1.22	2 × 10^−5^	rs35260072	0.476	260
		131779032	131788334	4.9e-24		0.235	20	131630852	0.524	
5	**Y_RNA**	ENSG00000202533	rs2548993	4.9e-09	**1.3 × 10** ^**−7**^	0.45	4 × 10^−9^	rs35260072	0.546	328
		131803894	131808869	1.2e-34		0.085	20	131630852	0.454	
5	**IRF1**	ENSG00000125347	rs11741255	7.0e-16	**5.0 × 10** ^**−10**^	−2.13	**0.052**	rs35260072	**0.952**	359
		131821895	131811182	1.6e-22		0.342	20	131630852	0.048	
5	**KIF3A**	ENSG00000131437	rs7731422	3.5e-08	**4.0 × 10** ^**−7**^	−0.76	0.007	rs35260072	5e-07	450
		132050825	132075653	4.1e-38		0.149	20	131630852	1.00	
6	**HLA-DRB5**	ENSG00000198502	rs9271055	1.1e-08	**1.2 × 10** ^**−8**^	−0.14	6 × 10^−6^	rs9274732	0.087	2866
		32491592	32575369	3.3e-310		0.024	20	32637825	0.913	
6	**HLA-DRB6**	ENSG00000229391	rs112112734	3.9e-18	**5.9 × 10** ^**−18**^	−0.23	6 × 10^−18^	rs9274732	0.546	2296
		32524144	32453853	3.3e-310		0.027	20	32637825	0.454	
6	**HLA-DRB1**	ENSG00000196126	rs9271470	3.5e-31	**5.2 × 10** ^**−30**^	−0.26	2 × 10^−11^	rs9274732	3e-04	1401
		32552085	32588662	3.3e-310		0.023	20	32637825	1.00	
6	**HLA-DQA1**	ENSG00000196735	rs1063355	9.7e-21	**3.2 × 10** ^**−20**^	−0.26	2 × 10^−29^	rs9274732	1e-07	1712
		32605397	32627714	3.3e-310		0.029	20	32637825	1.00	
6	**HLA-DQB1-AS1**	ENSG00000223534	rs1049225	1.9e-19	**5.9 × 10** ^**−19**^	−0.20	9 × 10^−25^	rs9274732	4e-16	1694
		32628081	32627747	3.3e-310		0.023	20	32637825	1.00	
6	**HLA-DQB1**	ENSG00000179344	rs1063355	9.7e-21	**1.9 × 10** ^**−20**^	−0.28	2 × 10^−14^	rs9274732	0.01	4028
		32631702	32627714	3.3e-310		0.030	20	32637825	0.99	
6	**HLA-DQA2**	ENSG00000237541	rs9271544	3.7e-31	**1.8 × 10** ^**−30**^	0.22	8 × 10^−6^	rs9274732	9e-05	1599
		32712055	32590120	3.3e-310		0.019	20	32637825	1.00	
6	**HLA-DQB2**	ENSG00000232629	rs1063355	9.7e-21	**5.2 × 10** ^**−20**^	0.30	8 × 10^−15^	rs9274732	9e-07	991
		32727593	32627714	3.3e-310		0.033	20	32637825	1.00	
6	**TAP2**	ENSG00000204267	rs115493740	5.6e-11	**1.1 × 10** ^**−9**^	−0.49	0.002	rs9274732	8e-34	609
		32798083	32838539	3.6e-61		0.080	20	32637825	1.00	
7	**FOXK1**	ENSG00000164916	rs10257680	3.5e-07	**4.6 × 10** ^**−7**^	−0.34	0.049	rs7781115	**0.996**	247
		4747231	4775507	3.8e-279		0.067	20	4784816	0.004	
17	**ALOX15**	ENSG00000161905	rs72835630	5.9e-07	**6.7 × 10** ^**−7**^	0.28	0.015	rs71368508	0.492	510
		4539893	4562449	3.3e-310		−0.262	20	4521473	0.508	
17	**ARRB2**	ENSG00000141480	rs55682338	1.2e-06	**1.4 × 10** ^**−6**^	−0.26	0.014	rs71368508	0.498	640
		4619289	4582183	3.3e-310		0.054	20	4521473	0.501	
19	**AXL**	ENSG00000167601	rs1709138	2.5e-10	**3.4 × 10** ^**−9**^	0.92	0.002	rs338593	0.069	143
		41746389	41719851	5.2e-62		0.156	20	41704304	0.931	

**FIGURE 1:**
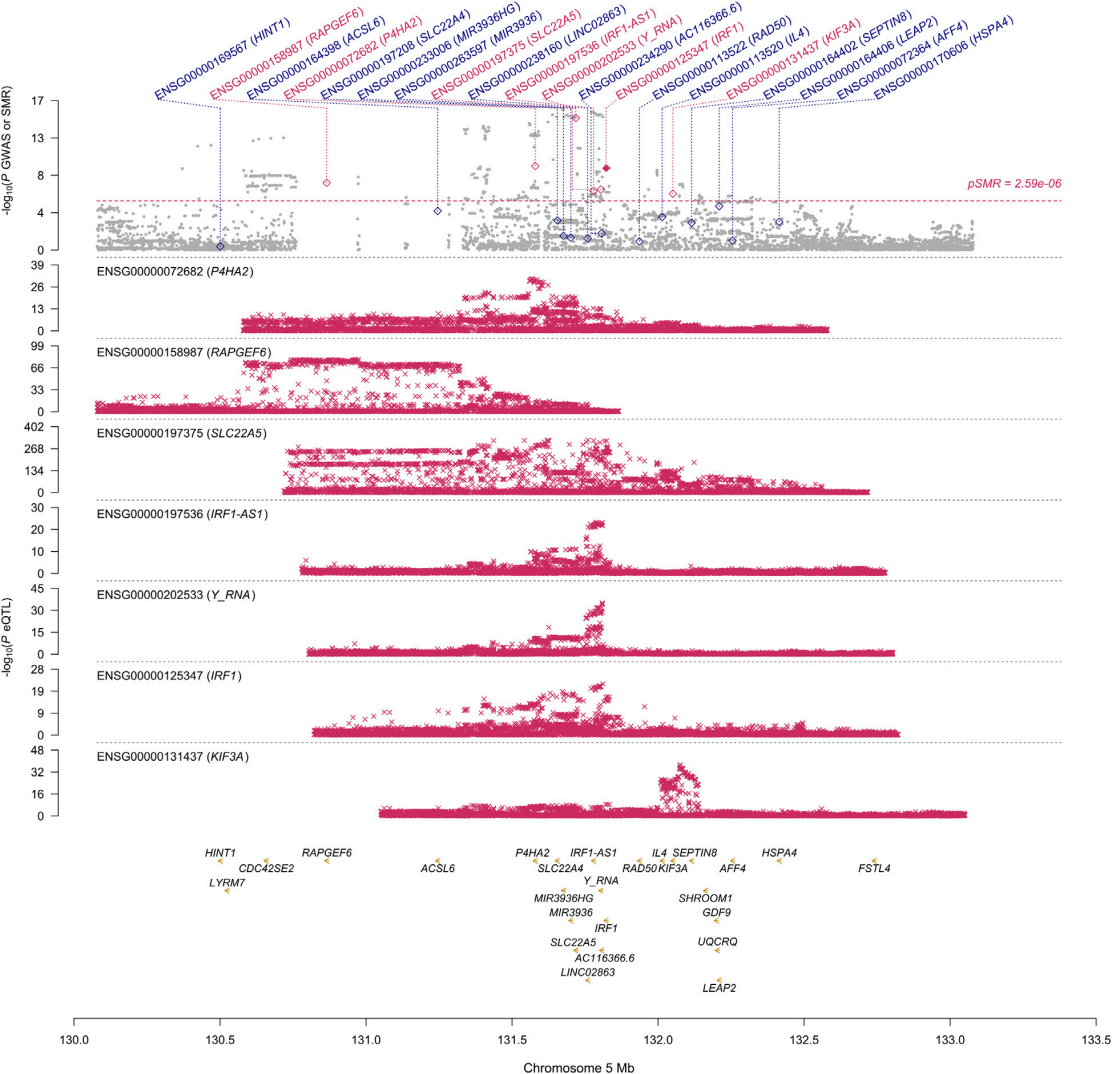
SMR locus plot for NP at the IRF1 locus using the blood eQTL data. In the top plot, each gray dot represents an SNV from the GWAS on NP. A red diamond shows that the probe has passed the SMR test, and the solid diamond shows that the probe has passed the HEIDI test as well. In the bottom plot, each red cross represents an SNV from the eQTL study for each gene. On the *x*-axis, the genomic positions (Mb, GRCh37) of SNVs, probes, and genes on chromosome 5 are shown. On the *y*-axis, −log10 *p*-values for SNVs from the GWAS on NP, SMR test, and eQTL study for IRF1 gene are shown.

First, we conducted the SMR analysis to integrate the GWAS and blood eQTL data to identify the most relevant genes whose expression in blood was significantly associated with the trait of NP. A total of 26 genes passed the SMR test (Figure 1, Supplementary Figures S1–S6). In chromosome 1, TNFRSF18 and CTSK genes passed both the SMR and HEIDI tests and thus were significantly associated with the trait of NP because of pleiotropy or causality (Supplementary Figures S1, S2). In particular, TNFRSF18 might be a “new candidate” that had no genome-wide significant SNV at *p* < 5 × 10^−8^ within 0.5 Mb of the probe (Supplementary Figure S1), as defined in the original SMR paper (Zhu et al., 2016). In chromosome 5, RAPGEF6, P4HA2, SLC22A5, IRF1-AS1, Y_RNA, IRF1, and KIF3A genes passed the SMR test. However, all genes but IRF1 failed the HEIDI test, suggesting that the associations between these six genes and the trait might be due to linkage (Figure 1). In chromosome 6, all the nine genes passed the SMR test but failed the HEIDI test (Supplementary Figure S3); however, the results must be interpreted with caution because of the complexity of LD patterns in the major histocompatibility complex (MHC) region (28.4–33.4 Mb on chromosome 6 based on GRCh37) (Zhu et al., 2016). In chromosome 7, FOXK1 gene failed the HEIDI test, but the *p*-value was marginal (Supplementary Figure S4).

**FIGURE 2:**
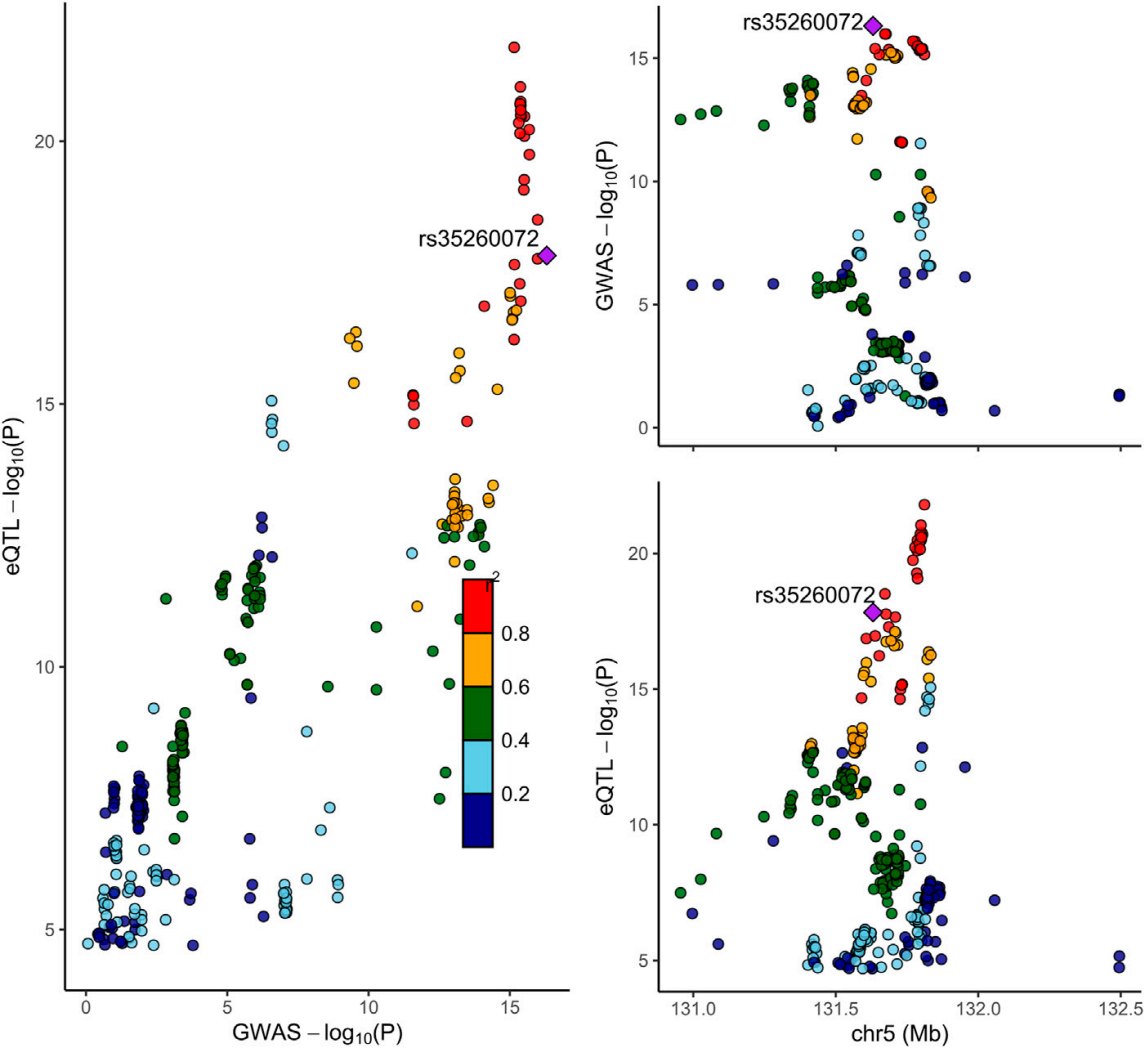
Locus compare plot for the COLOC analysis of SNVs associated with IRF1 expression in blood and NP. Each dot represents an SNV whose color indicates the LD (*r* ^2^) with the GWAS lead variant shown as a purple diamond. In the right panel, the genomic positions (Mb, GRCh37) on chromosome 5 are shown on the *x*-axis, and −log10 *p*-values for SNVs from the GWAS on NP (top) and eQTL study for IRF1 gene (bottom) are shown on the *y*-axis. In the left panel, the *p*-values of both the GWAS on NP and the IRF1 expression are compared.

Next, we conducted the COLOC analysis to integrate the GWAS and blood eQTL data of the genes that passed the SMR test and assess whether the genes were colocalized with the trait of NP. The COLOC test found strong support for colocalization between the trait and all the three genes (TNFRSF18, CTSK, and IRF1) that passed both SMR and HEIDI tests (Supplementary Figures S7A, B, Figure 2), and thus, we considered these genes to be highly prioritized for follow-up functional studies. FOXK1 gene was also supported strongly for colocalization (Supplementary Figure S7W).

### 3.2 SMR analysis using blood sQTL data

A genetic variant can affect not only messenger ribonucleic acid (mRNA) levels but also pre-mRNA splicing. The former variant is eQTL, and the latter is known as sQTL, which is another important mechanism of genetic regulation. In fact, only part of GWAS signals has been ascribed to cis-eQTL (Qi et al., 2022). Therefore, we conducted SMR analysis to integrate the GWAS data on NP and the sQTL data in blood. The result revealed that two probes (chr6:31540534:31541950:clu_28879:ENSG00000198563.13 and chr6:31540664:31541950:clu_28879:ENSG00000198563.13) in DDX39B gene passed both the SMR (P_SMR_ = 3.4 × 10^−7^ and 3.6 × 10^−7^, respectively) and HEIDI (P_HEIDI_ = 0.62 and 0.58, respectively) tests; however, the result must be interpreted with caution because they were located in the MHC region.

### 3.3 Enrichment analysis

To elucidate underlying biological mechanisms of the prioritized NP-associated genes, we performed an enrichment analysis using Metascape (Zhou et al., 2019). We submitted TNFRSF18, CTSK, IRF1, and FOXK1 to the web portal. The result suggested that TNFRSF18, CTSK, and IRF1 genes were involved in the biological process of “cellular response to cytokine stimulus” (GO:0071345, −log10 *p* = 4.26).

### 3.4 Mediation analysis

A majority of European cases with CRS with NP are characterized by type 2 inflammation with eosinophilia (Hulse et al., 2015; Hopkins, 2019). To investigate whether eosinophilia can mediate the effect of the prioritized genes on NP, we aimed to conduct mediation analysis (Moncla et al., 2022) by multivariable MR using genetically predicted eosinophil cell count as a covariate. First, we conducted a univariable two-sample MR analysis using CTSK expression as exposure and NP as an outcome. The F-statistic for each of the four IV SNVs was above 10, indicating that weak instrument bias was unlikely (Supplementary Table S2). In accordance with the SMR result, the IVW (multiplicative random-effects) result indicated that CTSK expression was significantly associated with NP risk (*p* = 0.0077) (Supplementary Table S3; Supplementary Figure S8). Although Cochran’s Q statistic indicated heterogeneity among IV SNVs, the MR-Egger intercept and MR-PRESSO global test detected no apparent horizontal pleiotropy. Second, we conducted a univariable two-sample MR analysis using CTSK expression as exposure and eosinophil cell count as an outcome. Among the three IV SNVs, rs6690662 was genome-wide and significantly associated with the outcome as well (Supplementary Table S2). Therefore, we excluded it from the analysis. Both the F-statistics for the other two IV SNVs were above 10. The IVW (fixed-effects) result revealed a significant effect of CTSK expression on increased eosinophil cell count (*p* = 0.036), although we could not conduct the sensitivity analyses except for Cochran’s Q statistic calculation due to the insufficient number of IV SNVs (Supplementary Table S3; Supplementary Figure S9). Finally, we conducted IVW multivariable MR analysis using CTSK expression and eosinophil cell count as exposures and NP as an outcome. The association between CTSK expression and NP was not significant after adjustment for eosinophil cell count (Supplementary Table S4). Taken together, although the number of IV SNP was only a few, these results suggest that the effect of CTSK expression on NP might be at least partly mediated by the effect of eosinophil cell count (Moncla et al., 2022). Because of the limited number of IV SNVs, we could not perform IVW multivariable MR analysis using TNFRSF18, IRF1, or FOXK1 expression as exposures.

## 4 Discussion

This study aimed to prioritize NP-associated genes by conducting SMR and COLOC analyses. These approaches are two of the established gene prioritization methods for post-GWAS analysis (Li and Ritchie, 2021). For example, the GIANT consortium conducted the SMR analysis alone to integrate their GWAS data on body mass index with 941 significant loci (*p* < 1 × 10^−8^) and eQTL data and prioritized 138 genes (Yengo et al., 2018). Hernández Cordero et al. (2021) identified three genes in lung tissue that were associated with COVID-19 hospitalization using the SMR and COLOC methods. In our study, the SMR and HEIDI tests identified TNFRSF18, CTSK, and IRF1 genes whose expression in blood was associated with NP because of pleiotropy or causality. The COLOC tests strongly suggested that the expression of the three genes in blood, in addition to FOXK1 gene, was associated with NP by shared causal variants. The results showed overall consistency and reproducibility between the two methods to some extent, and thus, TNFRSF18, CTSK, and IRF1 genes that passed all the SMR, HEIDI, and COLOC tests could be highly prioritized. ALOX15 and SLC22A5 genes whose associations with NP were suggested by other studies (Kristjansson et al., 2019; Yin et al., 2022) passed our SMR test but failed our HEIDI and COLOC tests; however, the corresponding *p*-value from the HEIDI test and PP.H4 were not so far from the significant thresholds. Moreover, the *p*-value of SLC22A5 gene from the SMR test was much smaller than that of IRF1 gene (Figure 1). Therefore, the function of the genes that passed our SMR test should be analyzed and validated in future studies even if they failed our HEIDI and/or COLOC tests.

Although the Metascape result suggested that the prioritized genes might be involved in the process of cellular response to cytokine stimulus, the underlying mechanisms by which they are associated with NP remain unclear. NP can be considered a subgroup of CRS; two main subgroups are CRS with NP and CRS without NP (Hopkins, 2019). CRS with NP may be not only mostly idiopathic but also part of genetic diseases, vasculitis, and immune disorders, such as central compartment atopic disease and allergic fungal rhinosinusitis, that are IgE-mediated allergic diseases triggered by allergens. A majority of European cases with CRS with NP are characterized by type 2 inflammation with eosinophilia and elevated levels of type 2 cytokines such as interleukin-5 and 13 (Hulse et al., 2015; Hopkins, 2019). Although we could not find a relevant literature report, our mediation analysis suggested that CTSK expression might have a causal effect on NP through eosinophilia. On the other hand, clinical and experimental literature reports have suggested that some of genes that our study prioritized may be associated with NP to some extent. The levels of CTSK (cathepsin K) protein, a lysosomal cysteine proteinase, were higher in nasal tissues from CRS subjects with NP than in those from non-CRS controls (Boruk et al., 2020), suggesting a possibility that CTSK expression could promote the progression of CRS with NP. TNFRSF18 (tumor necrosis factor receptor superfamily member 18) is also known as GITR (glucocorticoid-induced tumor necrosis factor receptor-related protein). GITR protein levels were higher in nasal tissues from non-eosinophilic CRS with NP subjects (a popular endotype in East Asia) than in those from eosinophilic CRS with NP subjects (a popular endotype in Europe) and controls; however, the protein levels did not differ significantly between the eosinophilic CRS with NP and control groups (Yao et al., 2020). Further studies would be required to examine the effect of TNFRSF18 gene on CRS with NP in the European population. We could not find a literature report regarding associations between NP and IRF1 (interferon regulatory factor-1) or FOXK1 (forkhead box K1) gene, but a study reported that IRF1 promoter polymorphisms that decreased the gene expression were significantly associated with increased serum IgE levels and risk of atopic sensitization measured by the skin prick test (Schedel et al., 2008). ALOX15 (arachidonic acid 15-lipoxygenase) expression was upregulated and accompanied by immunofluorescent colocalization with CCL26 expression that mediated eosinophil recruitment and activation at inflammatory sites in NP epithelial cells (Xu et al., 2021).

This study had several major limitations. First, the SMR method cannot distinguish pleiotropic genes from the causative genes. The association between prioritized genes and NP needs to be validated by follow-up functional studies. Second, we may have missed some important genes that have functional associations with NP, especially because of the small sample size of the GWAS on NP. Third, our SMR and COLOC analyses were based on populations of predominantly European ancestry. Moreover, NP are characterized by eosinophilic inflammation in the European subjects whereas by non-eosinophilic inflammation in the Asian subjects (Yao et al., 2020). Therefore, our findings are unlikely to be generalized to the non-European populations and ethnicities. Fourth, no eQTL data from nasal and/or paranasal sinus mucosa were available. Although eQTL effects in blood tissue with a large sample size can be used as a proxy for other relevant tissues (Zhu et al., 2016), further studies using eQTL data from nasal and/or and paranasal sinus mucosa would be warranted.

In conclusion, we could prioritize several genes associated with NP, including TNFRSF18, CTSK, and IRF1, by the SMR and COLOC analyses using the latest GWAS data with 34 genome-wide significant loci and blood eQTL data. However, follow-up functional studies must be conducted in future to validate the functional associations of the genes with the underlying disease mechanisms.

## Data Availability

The summary-level data of the GWAS on NP was publicly available from the FinnGen consortium (https://www.finngen.fi/en). The eQTL dataset was publicly available from the eQTLGen consortium (https://www.eqtlgen.org/). The sQTL dataset in SMR format was publicly available from the SMR software tool (https://yanglab.westlake.edu.cn/software/smr/#DataResource). The GWAS summary statistics for TNFRSF18, CTSK, IRF1, FOXK1 expressions from the eQTLGen consortium, and eosinophil cell count from the Blood Cell Consortium were available from the MRC IEU Open GWAS database (https://gwas.mrcieu.ac.uk/datasets/) (GWAS ID: eqtl-a-ENSG00000186891, eqtl-a-ENSG00000143387, eqtl-a-ENSG00000125347, eqtl-a-ENSG00000164916, and ieu-b-33, respectively). The codes for this study are available at https://github.com/myosh-tky/Masahiro-Yoshikawa/tree/main.
