# Supplementary figures and images for: Prioritization of nasal polyp-associated genes by integrating GWAS and eQTL summary data
Source: Front Genet. 2023 Jun 23;14:1195213. doi: 10.3389/fgene.2023.1195213 (PMC10326843; doi:10.3389/fgene.2023.1195213)

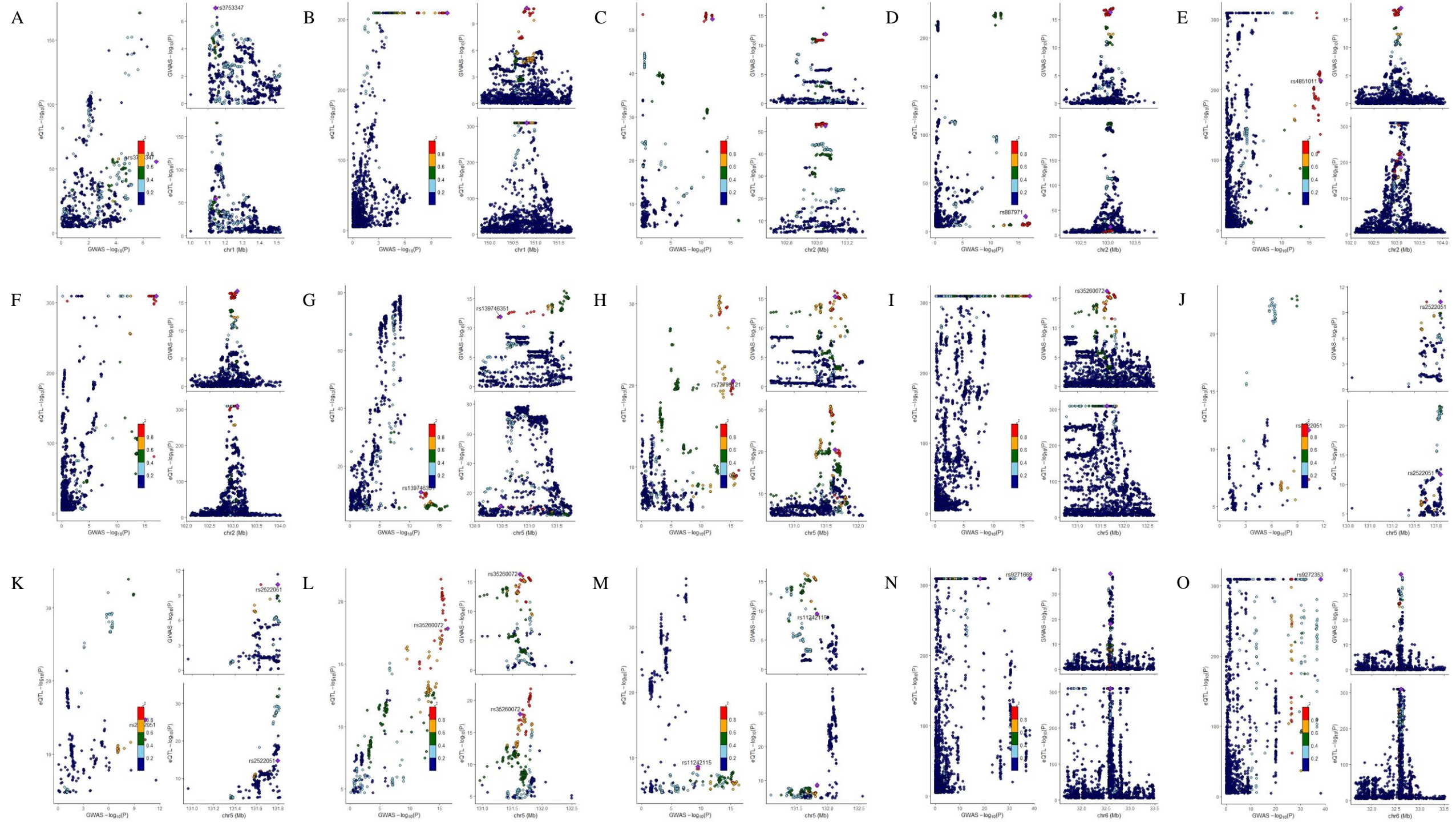

P

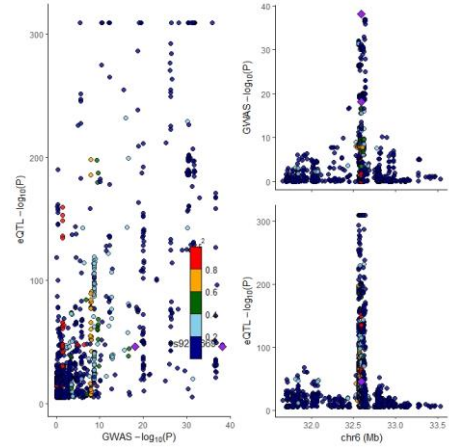

Q

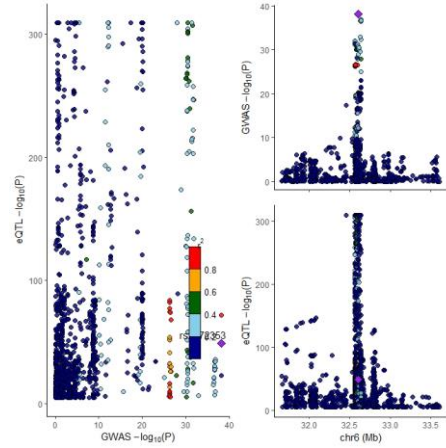

R

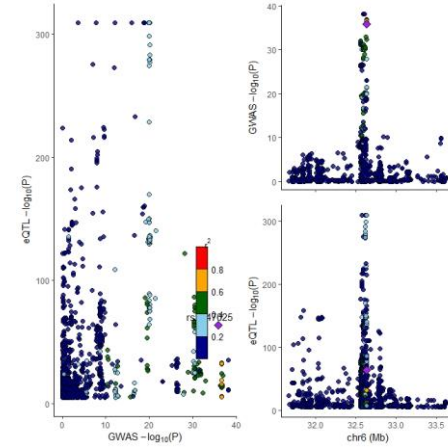

S

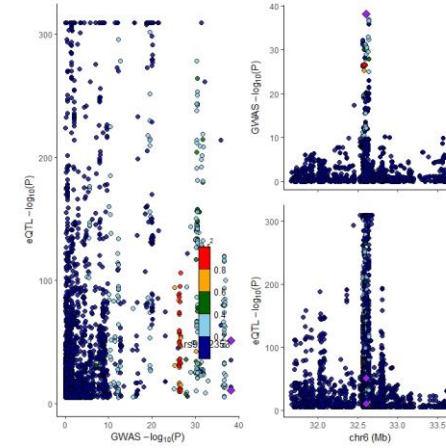

T

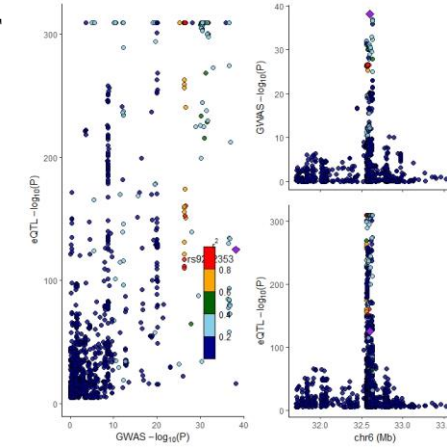

U

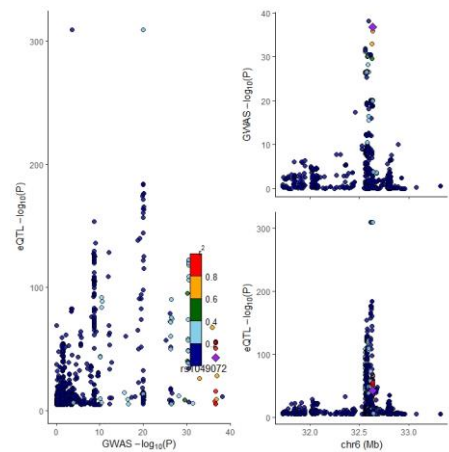

V

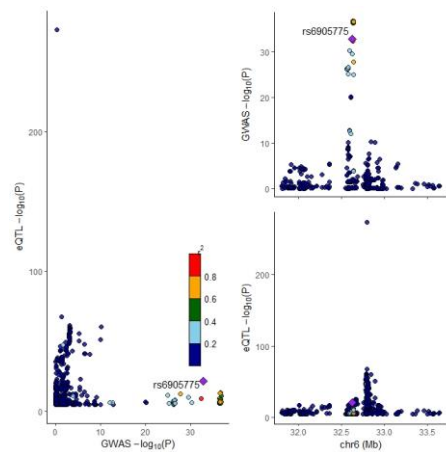

W

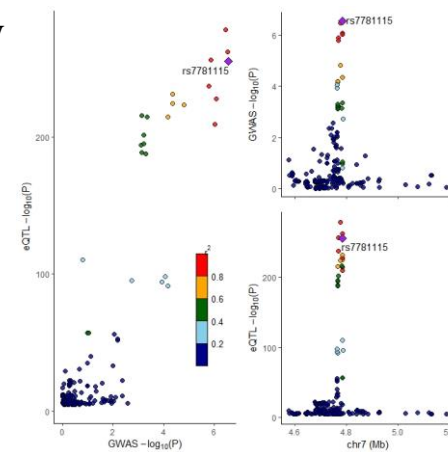

X

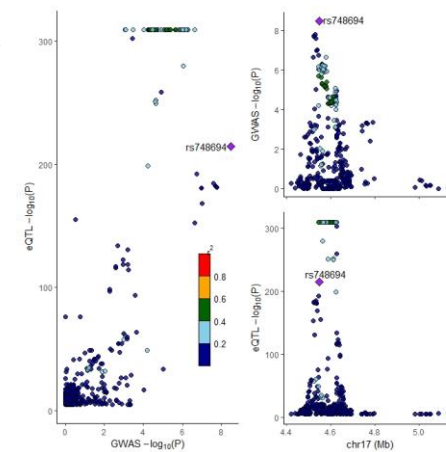

Y

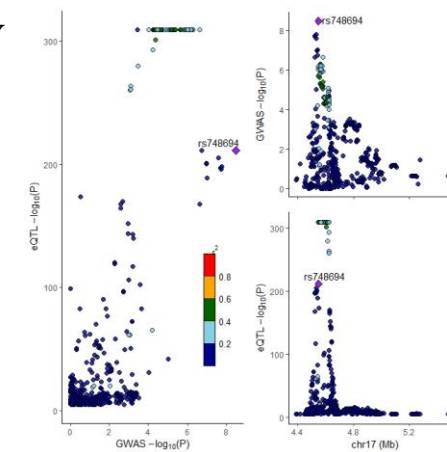

Z

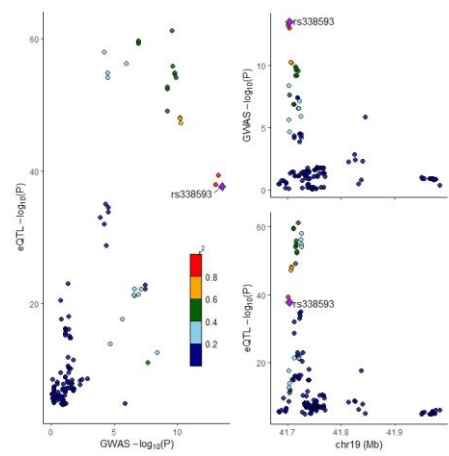

Supplement: Supplementary file 1 [file DataSheet7.PDF]

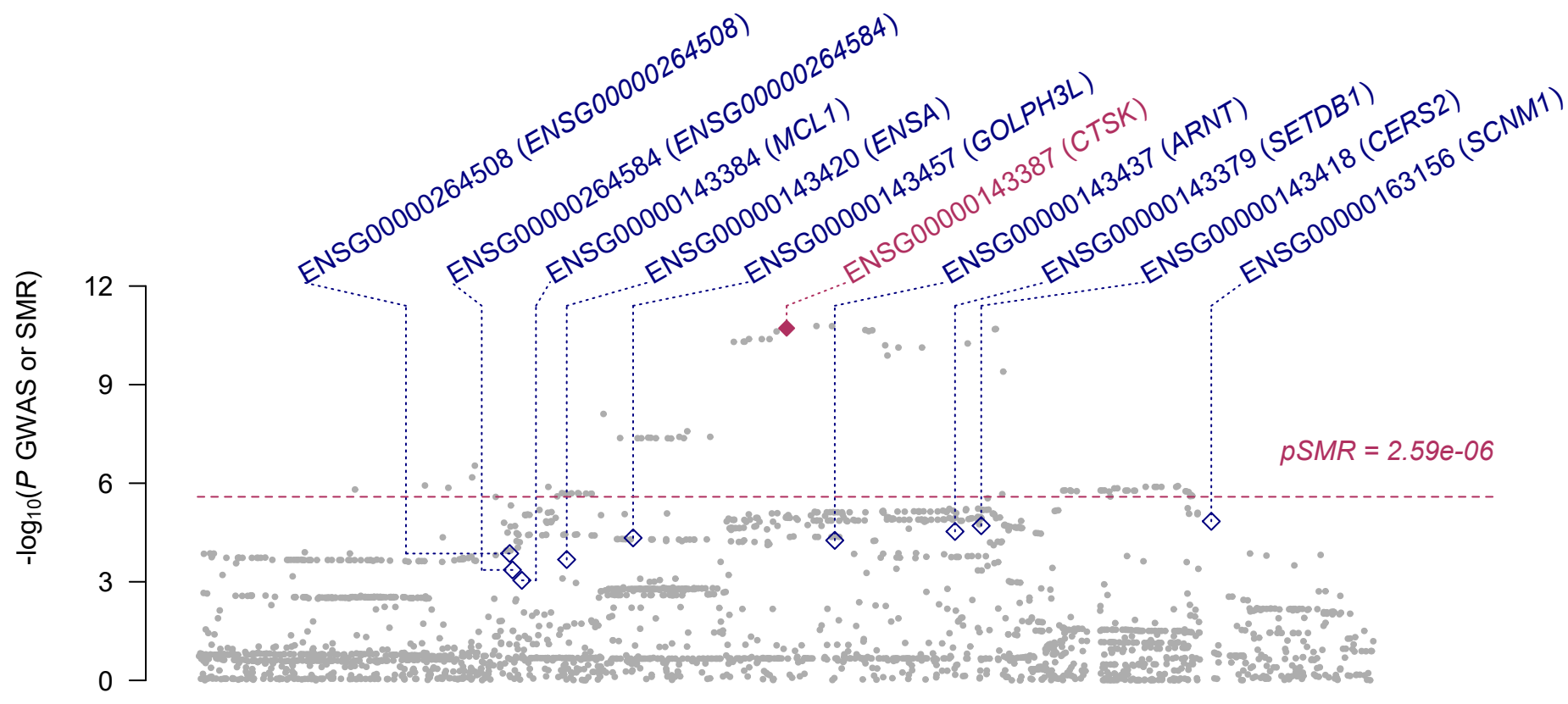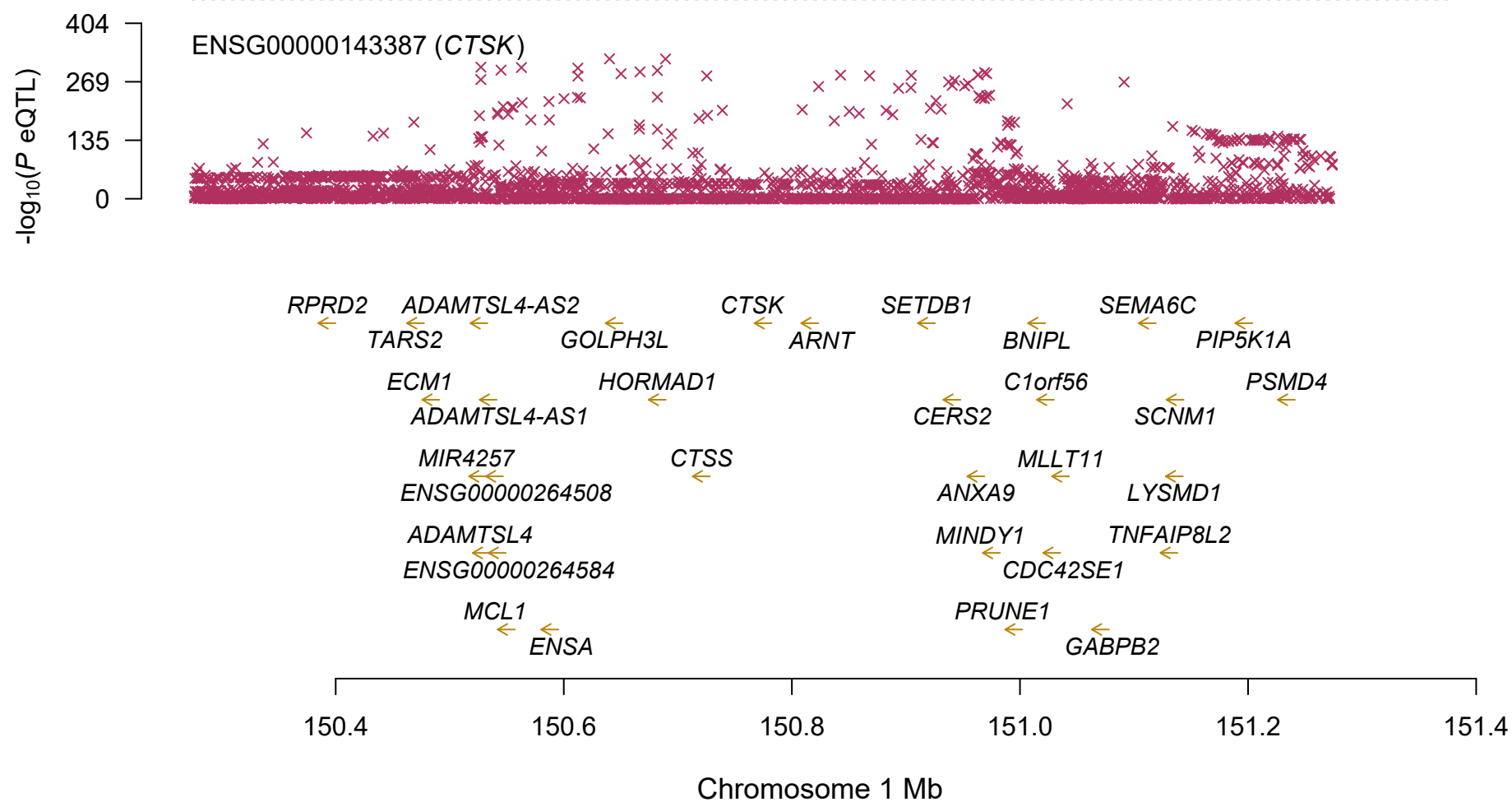

Supplement: Supplementary file 2 [file DataSheet2.PDF]

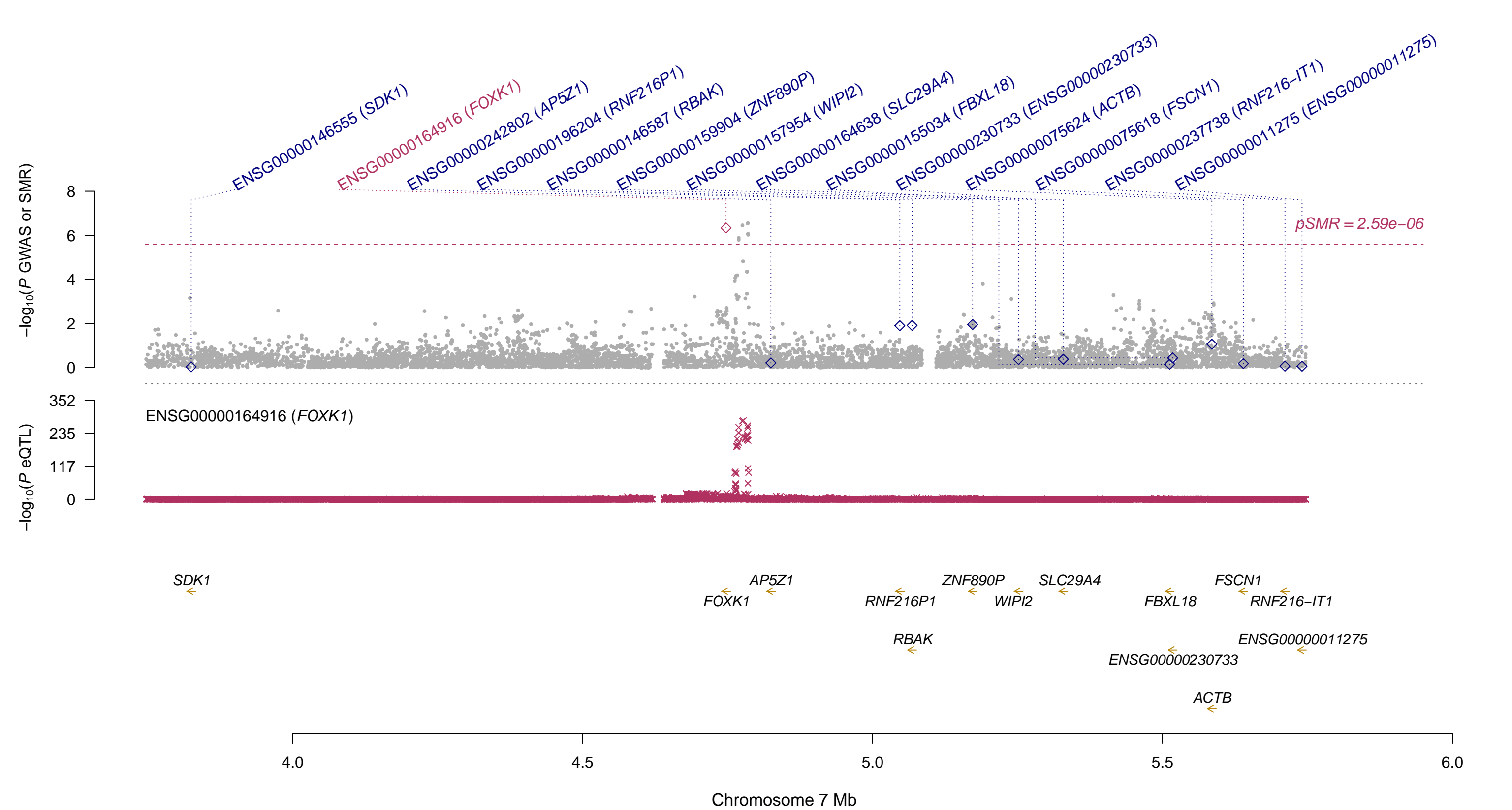

Supplement: Supplementary file 3 [file DataSheet4.PDF]

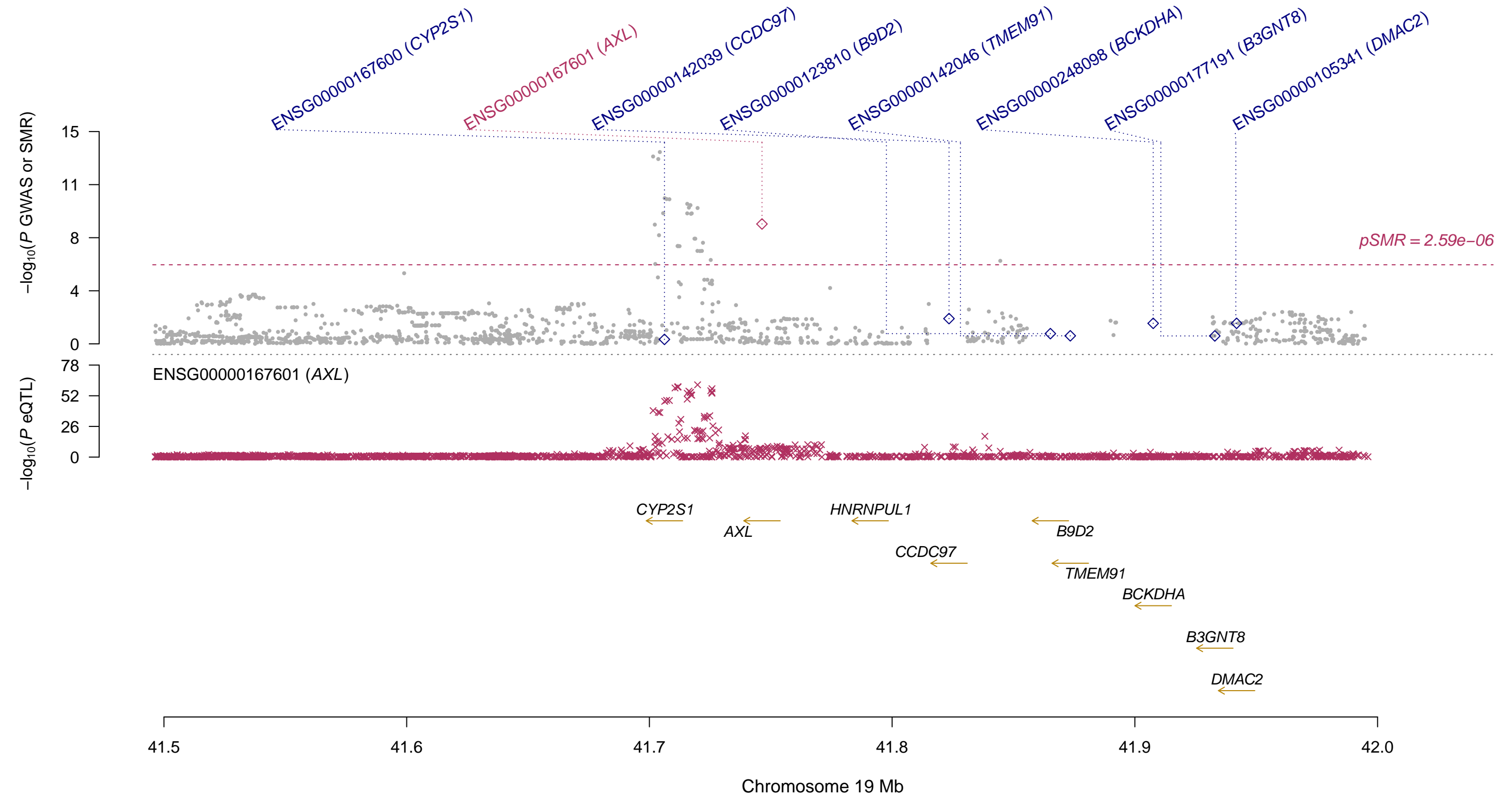

Supplement: Supplementary file 4 [file DataSheet6.PDF]

# MR Test

Inverse variance weighted (fixed effects)

SNP effect on eosinophil cell count || id:ieu-b-33

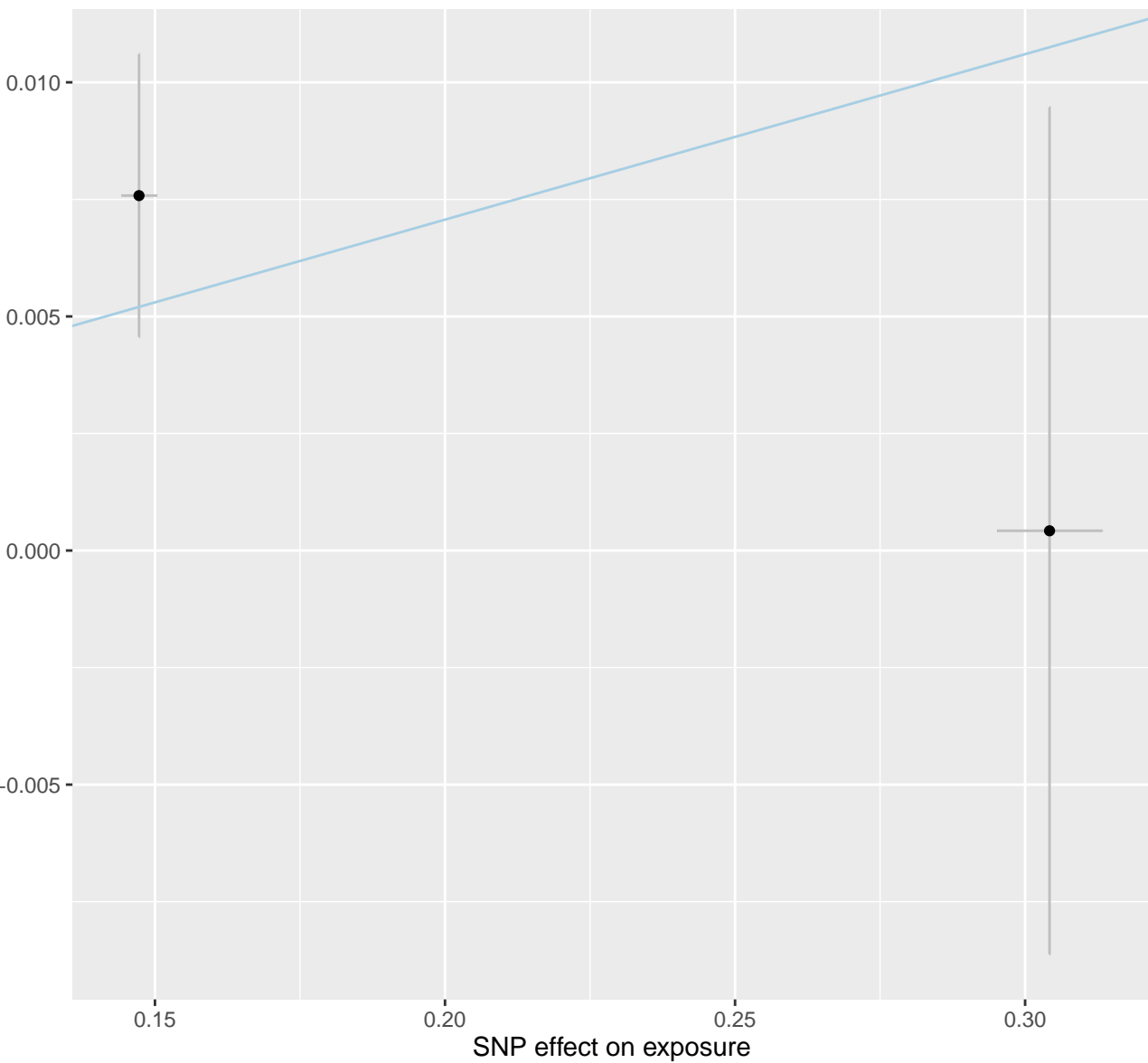

Supplement: Supplementary file 5 [file DataSheet9.PDF]

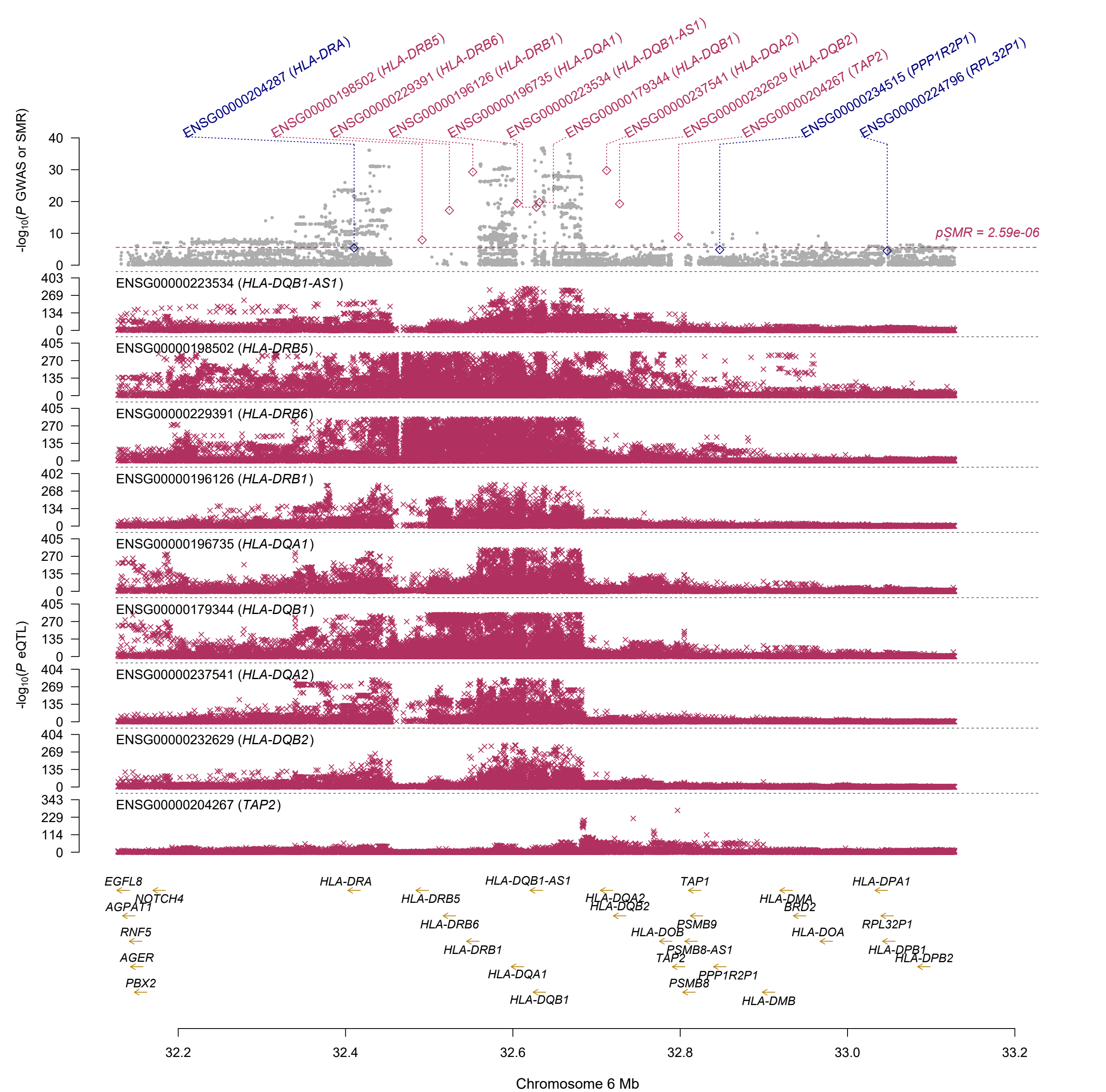

Supplement: Supplementary file 6 [file DataSheet3.PDF]

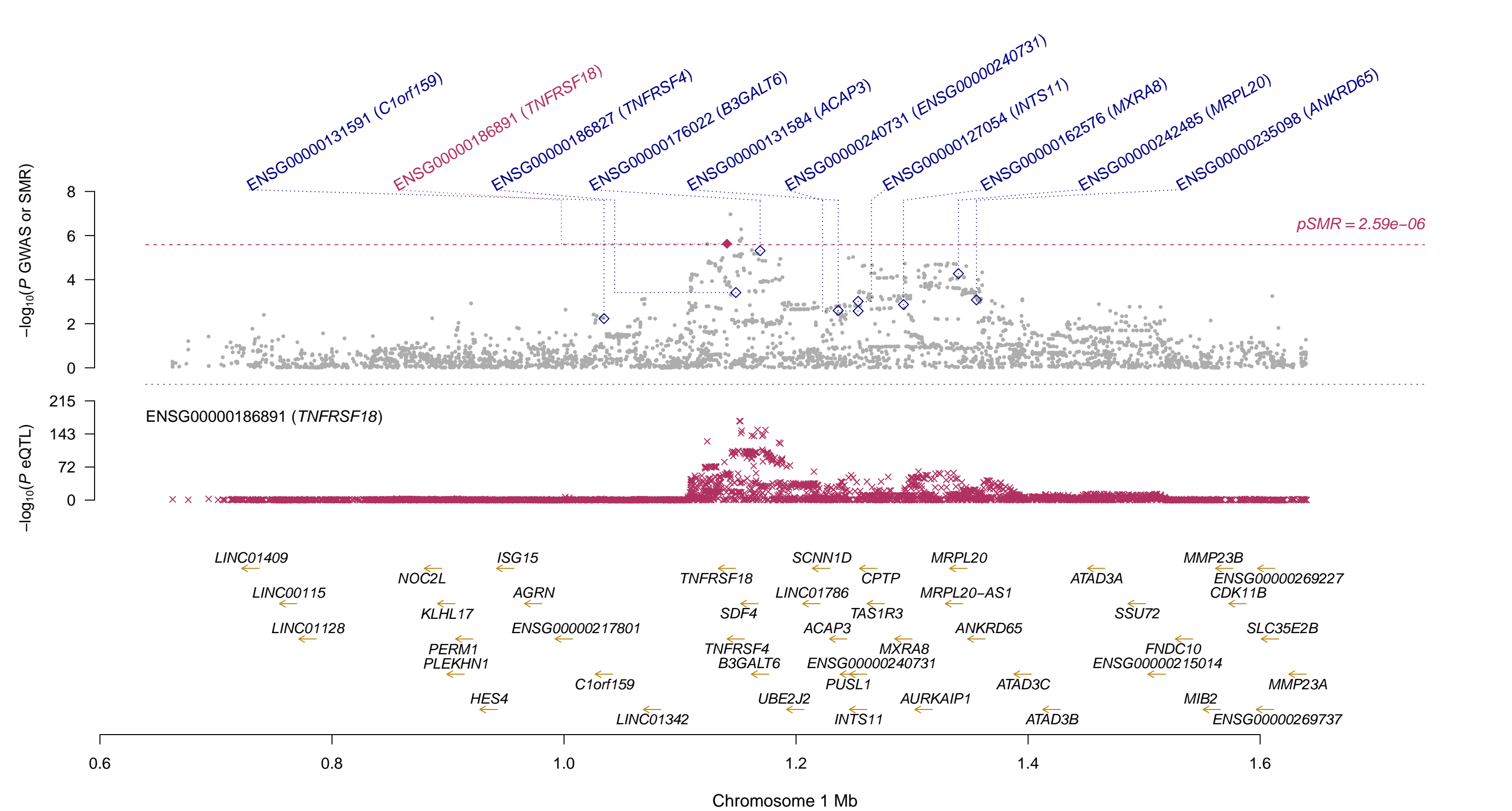

Supplement: Supplementary file 7 [file DataSheet1.PDF]

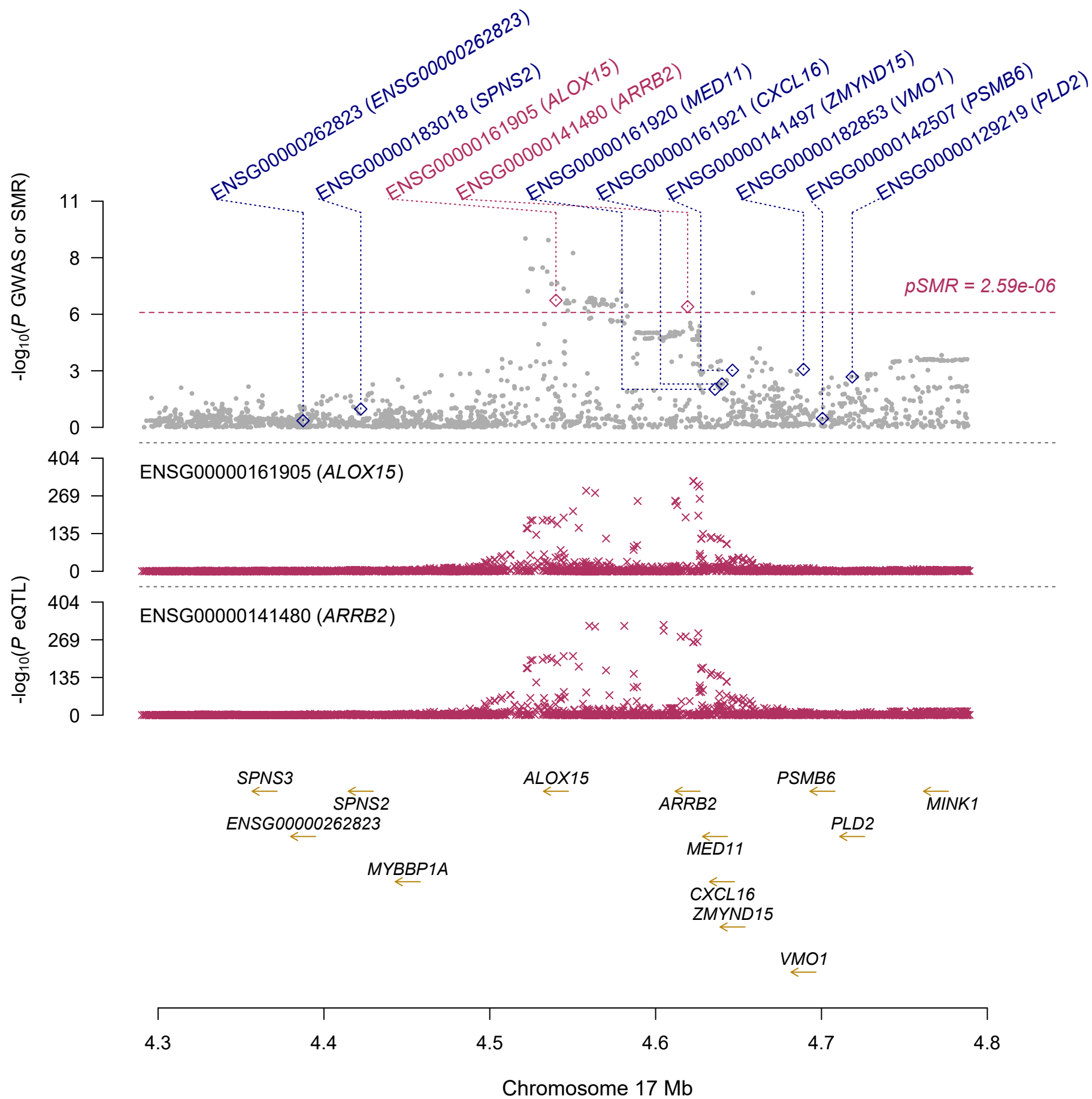

Supplement: Supplementary file 8 [file DataSheet5.PDF]
