# Supplementary material for: Prioritization of nasal polyp-associated genes by integrating GWAS and eQTL summary data
Source: Front Genet. 2023 Jun 23;14:1195213. doi: 10.3389/fgene.2023.1195213 (PMC10326843; doi:10.3389/fgene.2023.1195213)
Supplement: Supplementary file 10 [file DataSheet8.PDF]

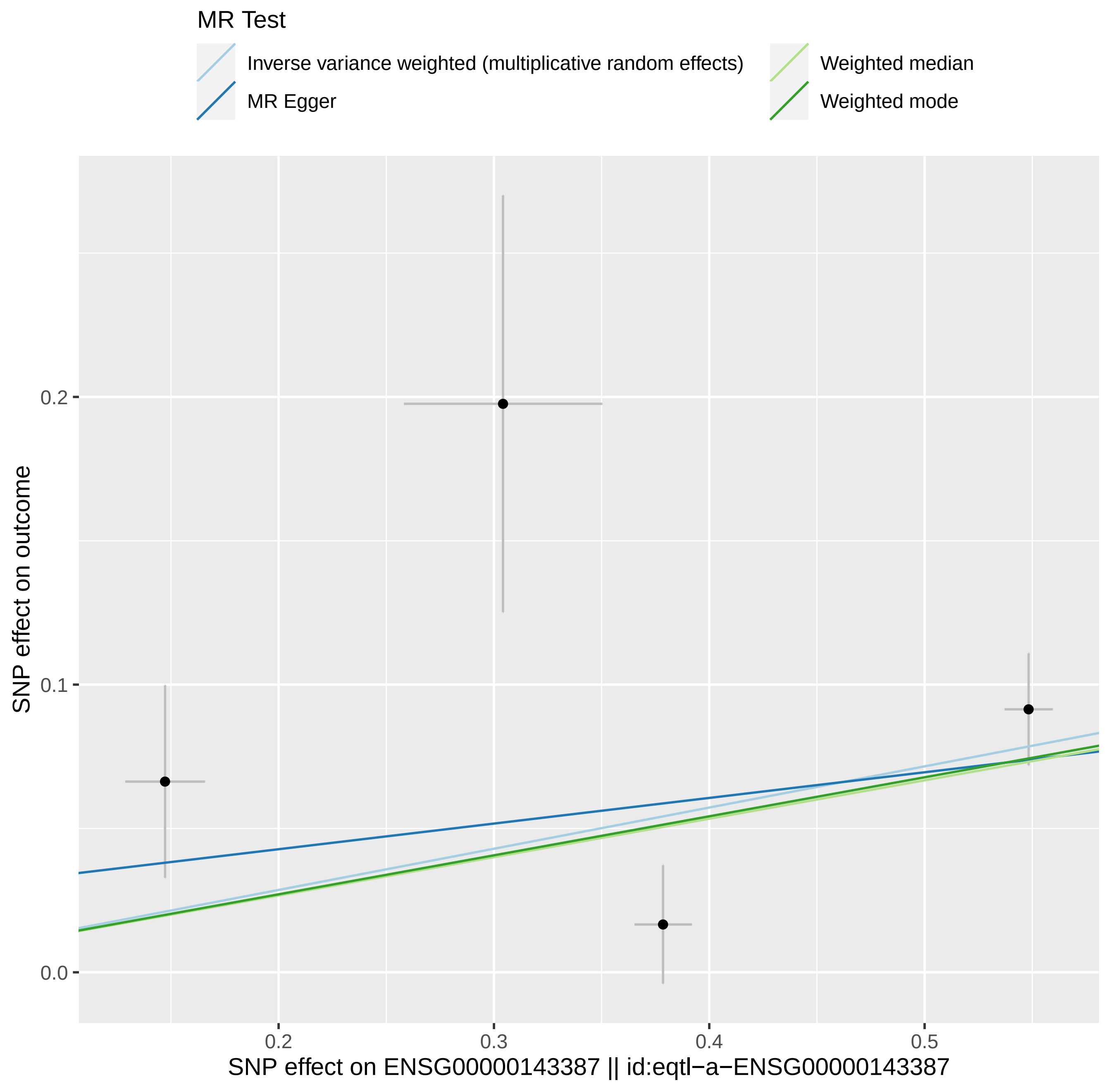
MR Test
Inverse variance weighted (multiplicative random effects)
MR Egger
Weighted median
Weighted mode
0.2
0.1
0.0
0.2
0.3
0.4
0.5
SNP effect on outcome
SNP effect on ENSG00000143387 || id:eqtl−a−ENSG00000143387
